# Microvesicle Formation Induced by Oxidative Stress in Human Erythrocytes

**DOI:** 10.3390/antiox9100929

**Published:** 2020-09-28

**Authors:** Julia Sudnitsyna, Elisaveta Skverchinskaya, Irina Dobrylko, Elena Nikitina, Stepan Gambaryan, Igor Mindukshev

**Affiliations:** 1 Center for Theoretical Problems of Physico-Chemical Pharmacology, Russian Academy of Sciences, Kosygina st., 4, 119991 Moscow, Russia; sudnitsyna@iephb.ru or; 2 Sechenov Institute of Evolutionary Physiology and Biochemistry, Russian Academy of Sciences, Thorez pr., 44, 194223 Saint-Petersburg, Russia; lisarafail@mail.ru (E.S.); dobrilko@mail.ru (I.D.); elena.nikitina@bk.ru (E.N.); s.gambaryan@klin-biochem.uni-wuerzburg.de (S.G.); 3 Center for Thrombosis and Hemostasis (CTH), University Medical Center of the Johannes Gutenberg-University Mainz, 55131 Mainz, Germany

**Keywords:** erythrocytes, microparticles, oxidative stress, vesiculation, band 3, *tert*-Bytyl hydroperoxide t-BOOH, nitric oxide donor, calcium ionophore A23187

## Abstract

Extracellular vesicles (EVs) released by different cell types play an important role in many physiological and pathophysiological processes. In physiological conditions, red blood cell (RBC)-derived EVs compose 4–8% of all circulating EVs, and oxidative stress (OS) as a consequence of different pathophysiological conditions significantly increases the amount of circulated RBC-derived EVs. However, the mechanisms of EV formation are not yet fully defined. To analyze OS-induced EV formation and RBC transformations, we used flow cytometry to evaluate cell esterase activity, caspase-3 activity, and band 3 clustering. Band 3 clustering was additionally analyzed by confocal microscopy. Two original laser diffraction-based approaches were used for the analysis of cell deformability and band 3 activity. Hemoglobin species were characterized spectrophotometrically. We showed that cell viability in *tert*-Butyl hydroperoxide-induced OS directly correlated with oxidant concentration to cell count ratio, and that RBC-derived EVs contained hemoglobin oxidized to hemichrome (HbChr). OS induced caspase-3 activation and band 3 clustering in cells and EVs. Importantly, we showed that OS-induced EV formation is independent of calcium. The presented data indicated that during OS, RBCs eliminated HbChr by vesiculation in order to sacrifice the cell itself, thereby prolonging lifespan and delaying the untimely clearance of in all other respects healthy RBCs.

## 1. Introduction

Extracellular vesicles (EVs), which consist of microvesicles (MVs), microparticles (MPs), and exosomes, are continuously produced in human blood from different cell types including circulating and endothelial cells. EVs contain various molecules of parent cells such as different proteins, bioactive lipids, and RNAs that can be taken up by recipient cells [1]. EVs are directly involved in different physiological processes such as vasoregulation, thrombosis, hemostasis, and inflammation, acting similar to signaling molecules or by direct transport of their constituents [2,3]. In normal conditions, red blood cell (RBC)-derived EVs compose 4–8% of all circulating EVs [4]. EV formation is triggered by structural alterations of the cell membrane, which are driven by factors that disrupt erythrocyte skeleton/membrane attachment, including aging-associated oxidative damage [5,6], reactive oxygen species (ROS) [7], increased intracellular Ca^2+^ concentration [8,9], adenosine triphosphate depletion [10], and RBC storage in blood banks [11]. RBC-derived EVs are not homogeneous by their size and content. The increase in intracellular calcium concentration induces membrane shedding, producing EVs containing cytoskeleton proteins, glycophorin, band 3 protein (band 3, anion transporter 1, AE1), and acetylcholine esterase, but not hemoglobin (Hb) [12]. During RBC concentrates storage, EVs carry aggregates of Hb, band 3, cytoskeleton proteins, caspases 3 and 8, Cluster of Differentiation (CD) 47, and immunoglobulins G [13]. Throughout the 120 days of RBCs’ lifespan, up to 20% of Hb is lost due to EV formation [14]. Formation of such EVs is proposed to rescue RBCs by getting rid of damaged Hb molecules and clustered band 3, which are well-defined markers of senescent RBCs, therefore prolonging the life span of these cells [15]. Two major mechanisms are described for EV formation including an increase in intracellular calcium concentration and oxidative stress (OS)-induced Hb oxidation [16]. Ca^2+^-activated protease calpain is involved in vesiculation by binding and degrading band 3, band 4.1, and ankyrin, which lead to alteration of cell deformability [9,16,17,18,19]. OS triggers membrane and Hb oxidation, clustering, and disruption of band 3-cytoskeleton anchorage, and all these alterations lead to EV formation [7,17,20,21,22,23]. All these data indicate that there are various mechanisms of RBC-derived EV formation, however, several questions are still unsolved. In this study, we elucidated the effects of different stimuli such as oxidant concentration and Hb oxidation state, as well as the role of calcium, caspase-3 activation, and band 3 clustering that trigger RBC-derived EV formation.

Here, we showed that cell viability in *tert*-Butyl hydroperoxide-induced OS directly correlated with the ratio of oxidant concentration to cell count, and that RBC-derived EVs contained Hb oxidized to hemichrome (HbChr). OS induced caspase-3 activation and clustering of band 3 protein in both, cells and EVs. Importantly, we showed that OS-induced EV formation was independent of calcium. Presented data indicated that during OS, RBCs eliminate HbChr by vesiculation in order to sacrifice the cell itself, thereby prolonging lifespan and delaying the untimely clearance of in all other respects healthy RBCs.

## 2. Materials and Methods

### 2.1. Reagents and Chemicals

Calcein-AM and eosin-5-maleimide (EMA) were from Molecular Probes (Eugene, OR, USA). Annexin-V conjugated with fluorescein isothiocyante (Annexin-V-FITC) was obtained from Biolegend (Amsterdam, Netherlands). Anti-active caspase-3 FITC polyclonal antibodies were from BD Pharmingen (San Diego, CA, USA). *tert*-Butyl hydroperoxide (t-BOOH), S-nitroso-L-cysteine (SNC)—sodium nitrite and L-cysteine hydrochloride monohydrate, calcium ionophore A23187, and basic buffer constituents were from Sigma-Aldrich (Munich, Germany). The buffers were isotonic with osmolality 300 mOsm/kg H_2_O controlled by cryoscopic osmometer Osmomat 3000 (Gonotec, Germany), pH 7.4, and had the following composition (in mM): HEPES buffer-NaCl, 140; KCl, 5; 4-(2-hydroxyethyl)-1-piperazineethanesulfonic acid, HEPES, 10; MgCl_2_, 2; *d*-glucose, 5; NH_4_^+^ buffer -NH_4_Cl, 140; KCl, 5; HEPES, 10; MgCl_2_, 2; *d*-glucose, 5. Ca^2+^ (2 mM) or ethylene glycol-bis(β-aminoethyl ether)-N,N,N′,N′-tetraacetic acid EGTA (2 mM) were added to HEPES buffer, as indicated.

### 2.2. Methods

#### 2.2.1. RBC Preparation

Human blood was collected from healthy volunteers, who did not take any medication at least 10 days before the experiments, in S-monovette tubes (9NC, Sarstedt, Nümbrecht, Germany) with the addition of 2mM EGTA. The donors provided their informed consents prior the blood draw, which was performed according to our institutional guidelines and the Declaration of Helsinki. Studies using human RBCs were approved by the Sechenov Institute of Evolutionary Physiology and Biochemistry of the Russian Academy of Sciences (IEPHB RAS) (study no. 3-03; 02.03.2019). RBCs were prepared by centrifugation of whole blood at 400× *g* (Centrifuge ELMI-50CM, Elmi, Latvia) in HEPES buffer with EGTA for 3 min at room temperature. Washed RBCs were resuspended in HEPES buffer with EGTA or Ca^2+^, as indicated, and adjusted to 0.5 × 10^9^ cells/mL (corresponding to Hematocrit 4.0–4.5%). The main blood parameters (red blood cell count and mean cell volume (MCV)) were controlled by the hematological counter Medonic-M20 (Boule Medical A.B., Stockholm, Sweden).

#### 2.2.2. Stress Models

Oxidative stress was induced by *tert*-Butyl hydroperoxide, t-BOOH (0.25, 0.5, 1, 1.5, 2 mM). Calcium stress, as increased intracellular Ca^2+^ concentration, was inducted by calcium ionophore A23187 (1 µM) in HEPES buffer with 2 mM calcium. Nitrosative stress was inducted by S-nitroso-L-cysteine, SNC (500 µM). To evaluate whether t-BOOH effects on RBCs are reversible or not, we incubated RBCs with 1 or 2 mM of t-BOOH for 0.5 h; the cells were divided into two groups, one of which was washed 2 times by HEPES buffer with EGTA (“washed”), and the other left without washing out t-BOOH (“no washing”).

#### 2.2.3. Microparticle Isolation and Analysis

In this study, we investigated EVs and divided them by their size, as characterized by flow cytometry, and content, as characterized by spectrophotometry, to MPs, which were smaller and did not contain Hb, and MVs, which were bigger and contained Hb. MP/MV isolation was performed according to the protocol described in detail in [24]. Briefly, for analysis of MPs/MVs, we incubated RBCs with the indicated compounds for 24 h, then the samples were gently centrifuged (50× *g*, 7 min) and the supernatant was collected for the future analysis. RBC supernatant was centrifuged at 20,000× *g* for 30 min (centrifuge 5810R, Eppendorf, Hamburg, Germany) for separation of free Hb and pellet-containing MVs and MPs. The presence of MVs and MPs in pellets was confirmed by flow cytometry, and then MVs and MPs were scanned spectrophotometrically for Hb species analysis.

#### 2.2.4. Spectral Analysis of Hemoglobin Species

Absorption spectra of Hb species were registered by spectrophotometer SPECS SSP-715-M (Spectroscopic Systems LTD, Moscow, Russia) in the wavelength range of 300–700 nm with a step size of 1 nm at 25 °C. To study the effects of different stresses on free Hb, we hypoosmotically lyzed intact cells and added the indicated compounds, and then the spectra were collected at the indicated time. To study the Hb transformation in cells, we incubated the RBCs with the indicated compounds for the indicated time, and then the cells were hypoosmotically lyzed and the free Hb spectra were scanned. To study the Hb species encapsulated in MPs/MVs, we isolated MPs/MVs, as described in Section 2.2.3, and scanned them.

##### Hemoglobin Species Calculation

The percentage of oxidized Hb in RBC suspensions was determined by spectrophotometry using the millimolar extinction coefficients of the different Hb species (oxyhemoglobin, oxyHb; methemoglobin, metHb; hemichrome, HbChr) according to [25]. Briefly, RBC lysates were scanned from 500 to 700 nm while recording the absorbance values at 560, 577, 630, and 700 nm. These data were used for the calculation of Hb species percentage using the equations presented in [25]. The data are presented as percentage from the sum of all Hb species in the sample taken as 100%.

##### Induction of Hypoxia

RBC suspension was degassed with argon for 15 min. The oxygen sensor mini-Oksik 3 (“Analitika service” Ltd, Moscow, Russia) was used to control the oxygen content in the hypoxia chamber (Billups-Rothenberg, San Diego, CA, USA), with absorption registered in the range of 300–700 nm. The cuvette was sealed up with wrapping film during the registration of absorption for maintenance of hypoxic conditions.

#### 2.2.5. Characterization of RBC Deformability by Laser Diffraction Method

To estimate the osmotic and ammonium fragility of RBCs, we used the novel laser diffraction method (laser microparticle analyzer LaSca-T, BioMedSystems Ltd., Saint-Petersburg, Russia), adapted for cell physiology, according to Mindukshev et al. [26,27,28]. The intensity of scattered light was continuously detected by forward scattering at various angles (Figures S1 and S2). The MCV data from hematological counter Medonic-M20 (Boule Medical A.B., Stockholm, Sweden) were used as initial volume values MCV_300_ for the calculation of the MCV changes by the original software of the laser particle analyzer LaSca-TM.

##### Osmotic Fragility Test (OFT)

RBCs (0.5 × 10^9^ cells/mL) were incubated at indicated concentrations of A23187, SNC, and t-BOOH at indicated times. Then, aliquots (10 µL) of each sample were resuspended in 1 mL of HEPES buffer for osmotic fragility test. Hemolysis curves were registered for a range of osmolality from 210 to 70 mOsm/kg H_2_O. For each osmolality step, we added corresponding volume of water and RBCs to the sample to keep RBC concentration constant. The cell volume investigation algorithm was used for the estimation of cell volume changes dynamics and percentage of hemolysis [26,27,28,29]. The following parameters were calculated from hemolysis curves: H50, an osmotic fragility variable that represents the saline concentration that induces 50% lysis; W, the distribution width; and MCV_120_, maximal mean cell volume during the OFT, which is calculated from the hemolysis curve based on MCV_300_. MCV_300_, mean cell volume, was controlled by a hematological analyzer. The basic principles of the OFT are described in Figure S1.

##### Ammonium Stress Test (AST)

RBCs were prepared as for OFT. Control RBCs (10^6^ cells/mL) were suspended in 1 mL of HEPES buffer with EGTA and then for AST in 1 mL of NH_4_^+^ buffer. The following parameters were calculated from the hemolysis curves: V_hem_, maximal hemolysis rate; %Hem, percentage of lyzed cells; and MCV_hem_, maximal mean cell volume during the AST, which is calculated from the hemolysis curve based on MCV_300_. The basic principles of the AST are presented in Figure S2.

#### 2.2.6. Flow Cytometry Analysis

All flow cytometry experiments were performed on flow cytometer Navios (BeckmanCoulter, Brea, CA, USA) with an analysis of no less than 20,000 events.

##### Size and Structure Analysis

For size and structure analysis, we used forward scatter (FSC)/side scatter (SSC) mode, which provides information about cell size and structure. The intensity of light scattered in a forward direction (FSC) correlates with cell size. The intensity of scattered light measured at a right angle or side scatter (SSC) correlates with internal complexity, granularity, and refractiveness [30,31,32].

##### Esterase Activity Analysis

Calcein-AM was used for the evaluation of cell esterase activity. RBCs (0.5 × 10^7^ cells/mL) were incubated with calcein-AM (5 μM, 40 min, 37 °C) then diluted in 300 µL HEPES buffer with the following registration of calcein fluorescence at green detector (fluorescence light sensor 1, FL1) by flow cytometer Navios (BeckmanCoulter, Brea, CA, USA).

##### Phosphatidylserine (PS) Externalization at the RBC Surface

Annexin-V is a Ca^2+^-dependent dye, and thus 2 mM Ca^2+^ was added to HEPES buffer for the annexin test. RBCs (0.5 × 10^9^ cells/mL) were incubated with annexin-V (0.1 µg/mL, 15 min, 25 °C), and then fluorescence of annexin was registered at FL1 by flow cytometry.

##### Analysis of Band 3 Clustering

The contribution of the cytoskeleton in RBC transformations under OS was estimated using the eosin-5-maleimide (EMA)-binding test. RBCs (0.5 × 10^9^ cells/mL) were incubated with EMA in the HEPES buffer (0.07 mM, 40 min) with the following registration of EMA fluorescence intensity at FL1 by flow cytometry.

##### Microparticle Detection

MPs and MVs were isolated as described in Section 2.2.3. The combined range of the cytometer optical system was used for data collection W2 (Wide-High 9°–19° + Narrow 2°–9° mode). For W2 FSC detection, we used Ultra Rainbow Fluorescent Particles at nominal size 3.0–3.4 µm (BD Biosciences, San Jose, CA, USA) and Latex Beads of 0.5 µm (BeckmanCoulter, Brea, USA) to estimate MP/MV sizes.

##### Caspase-3 Activation

RBCs at 0.5 × 10^9^ cells/mL were incubated with the indicated concentrations of A23187, SNC, and t-BOOH at indicated time, fixed in 1% formalin (10 min), and were then centrifuged (400× *g*, 3 min). Pellets then were resuspended in phosphate buffered saline containing 1% bovine serum albumin (PBS BSA 1%) and permeabilized by 1% Tween-20 for 10min. Anti-active caspase-3 FITC polyclonal antibodies were added to the samples and incubated for 20 min in the dark. Caspase-3 activity was registered as the mean fluorescence intensity increase at FL1.

#### 2.2.7. Confocal Microscopy

A Leica TCS SP5 MP scanning confocal microscope (Leica Microsystems Inc., Bannockburn, IL, USA) was used for evaluation of EMA-binding/band 3 clustering and MP and MV formation. Erythrocytes were captured with 20 (HCX APO CS 20/0.70; Leica Microsystems, Inc., Buffalo Grove, IL, USA) or 63 (HCX APO CS 63/1.4; Leica Microsystems, Inc., Buffalo Grove, IL, USA) immersion objectives. To resolve fine details (clustering and MVs), we used an additional electronic zoom with a factor of 1.5 to 3.5. For imaging, the emitted fluorescence was acquired at 500 to 600 nm (green region of the spectrum for EMA). Single focal plane images were merged and analyzed with standard Leica LAS AF Software (Leica Microsystems, Inc., Buffalo Grove, IL, USA).

#### 2.2.8. Data Analysis

Laser diffraction data were analyzed by the original software LaSca_32 v.1498 (BioMedSystems Ltd., Saint Petersburg, Russia) of the laser particle analyzer LaSca-TM. Flow cytometry data were analyzed by original software Cytometry List Mode Data Acquisition and Analysis Software v.1.2 for Navios cytometer (BeckmanCoulter, Brea, CA, USA) and by FCS Express Flow 7 (De Novo Software, Pasadena, CA, USA). Differences between groups were analyzed by IBM SPSS Statistics v.26 (IBM Corporation, Armonk, NY, USA). The data are presented as the mean ± SD. Our variables conformed to a normal distribution (Shapiro–Wilk’s test, *p* > 0.05). We used one-way analysis of variance (ANOVA) for group comparisons. When the samples were homoscedastic (Levene’s test, *p* > 0.05), we used Tukey honestly significant difference (HSD) post hoc analysis. When the equal variances were not assumed, we used Tamhane’s T2 post hoc analysis. For paired groups analysis, we used a paired *t*-test. *p* < 0.05 was considered statistically significant.

## 3. Results

### 3.1. RBC Viability Strongly Depended on the Ratio of Oxidant to Cell Count

**Figure 1:**
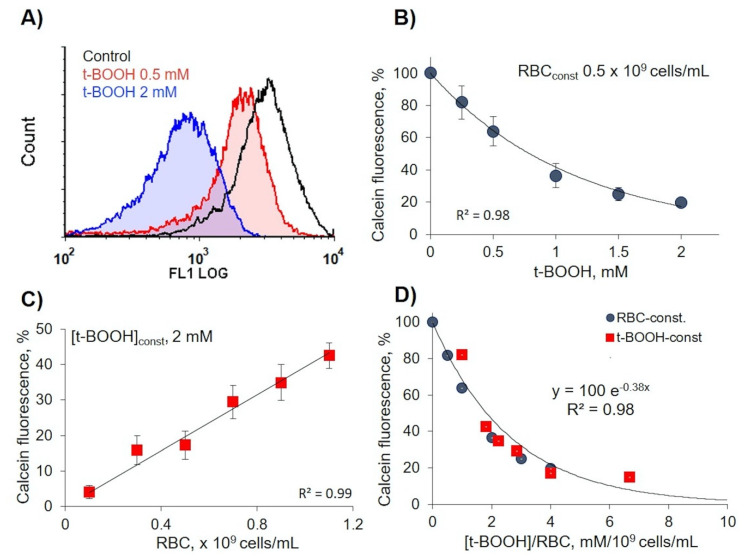
Calcein fluorescence intensity strongly depended on oxidant to cell count ratio. Red blood cell (RBC) suspension was incubated with *tert*-Butyl hydroperoxide (t-BOOH) at indicated concentrations for 1 h, then RBCs were stained with calcein-AM (5 µM, 40 min) and analyzed for calcein fluorescence intensity by flow cytometry at FL1 (logarithmic scale). (**A**) Representative histograms from five independent experiments. (**B**) Dependence of calcein fluorescence intensity in constant RBC count from t-BOOH concentrations. (**C**) Dependence of calcein fluorescence intensity in constant t-BOOH concentration from RBC count. (**D**) Exponential dependency between calcein fluorescence intensity and the ratio of oxidant concentration/cell count ([t-BOOH]/RBC). Data in (**B**,**C**) are presented as means ± SD, *n* = 5.

Calcein-AM could be used for the evaluation of RBC viability and OS-induced cytotoxicity [33]. Therefore, in our experiments, we used this test to assess the effects of OS on RBC viability through esterase activity changes. In constant RBC concentration (0.5 × 10^9^ cells/mL), calcein fluorescence intensity significantly decreased with t-BOOH concentration increase (Figure 1A,B), whereas in constant t-BOOH concentration, calcein fluorescence intensity directly correlated with RBC count (Figure 1C). Analysis of these two plots (Figure 1B,C) revealed exponential dependency (*R^2^* = 0.98) between calcein fluorescence intensity, as a marker of OS, and the ratio of oxidant concentration to cell count ([t-BOOH]/RBC) (Figure 1D). Here, we showed that it is very important to consider the ratio ([t-BOOH]/RBC) for characterization of OS effects on RBCs. On the basis of the presented data for all our experiments, we kept RBC concentration (0.5 × 10^9^ cells/mL) constant.

### 3.2. t-BOOH Induced RBC Vesiculation

**Figure 2:**
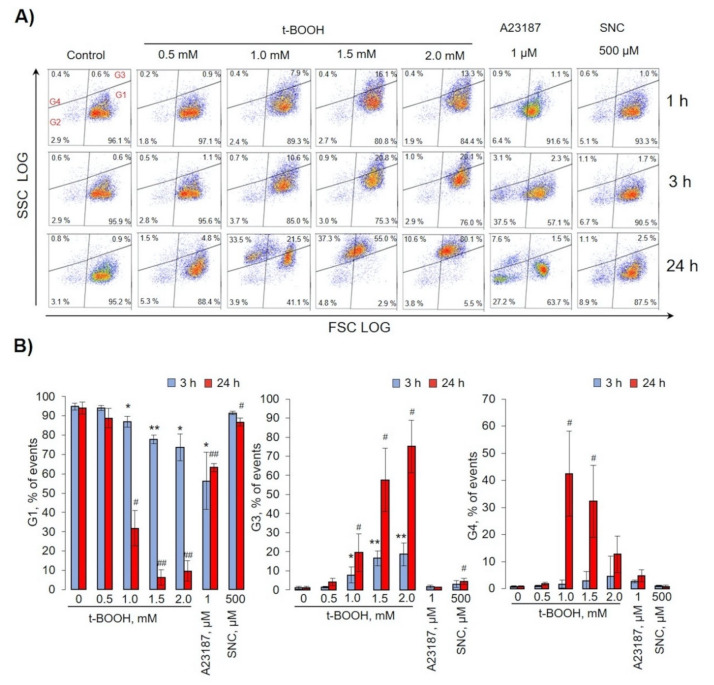
Oxidative stress induced by t-BOOH-triggered RBC transformations and microvesicle formation. RBCs (0.5 × 10^9^ cells/mL) were incubated at indicated concentrations of t-BOOH, A23187, and S-nitroso-L-cysteine (SNC) at the indicated times. Gate 1 represents control RBCs, gate 2—microparticles (MPs), gate 3—transformed RBCs, and gate 4—microvesicles (MVs). (**A**) Representative dot plots out of six independent experiments. (**B**) Quantification of presented data expressed as mean ± SD, *n* = 6. One-way ANOVA, Tamhane T2 (G1, G2 24 h, G4), or Tukey HSD post hoc (G2 3 h) were used where appropriate. * *p* < 0.05, ** *p* < 0.001 compared to control (t-BOOH 0mM, 3 h); # *p* < 0.05, ## *p* < 0.001 compared to control (t-BOOH 0mM, 24 h).

**Figure 3:**
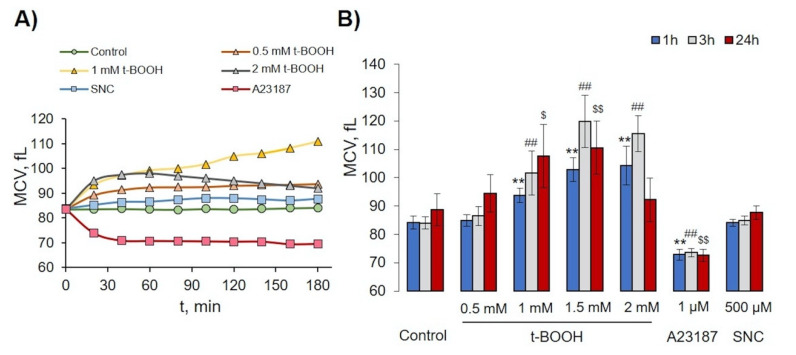
Effects of t-BOOH, A23187, and SNC on RBC volume changes. RBCs (0.5 × 10^9^ cells/mL) were incubated with indicated concentration of t-BOOH, A23187, and SNC for indicated times and were analyzed by hematological analyzer. (**A**) Representative histogram of mean cell volume (MCV) changes for one donor for 3 h; symbols indicate time of analysis. (**B**) Quantitative data from 10 independent experiments (10 donors). Data are presented as mean ± SD (*n* = 10), one-way ANOVA, Levene’s test < 0.05, and Tamhane T2 post hoc. ** *p* < 0.001 compared to 1h control; ##, *p* < 0.001 compared to 3 h control; $, *p* < 0.05, $$, *p* < 0.001 compared to 24 h control.

For analysis of RBC transformations, we used flow cytometry protocol according to our previous template [28], and cell volume changes were analyzed by the hematological analyzer (Figure 2 and Figure 3). For the evaluation of different types of RBC transformations, we divided dot plots for four regions. Gate 1 represents the native cells, gate 2—MPs, gate 3—transformed cells with increased SSC values, and gate 4—MPs with increased SSC values (referred to as microvesicles, MVs) (Figure 2).

In the control, more than 96% of RBCs were in gate 1 and remained constant for 24 h. t-BOOH for 1- and 3-h slightly increased the FSC (cell volume) concentration-dependently and increased SSC values (structural heterogeneity) without an increase in MP formation. After 24 h of 0.5 mM t-BOOH application, cells slightly increased SSC and FSC values, whereas 1 and 1.5 mM led to the formation of MVs and cells with increased SSC (gates 3 and 4). We also found that 2 mM of t-BOOH transformed RBCs to high SSC (gate 3). The increase of intracellular calcium concentration (facilitated by A23187) time-dependently triggered significant MP formation (gate 2), up to 32 ± 3% (*p* < 0.05, *n* = 6) after 24 h of incubation, whereas no significant MP formation triggered by t-BOOH or SNC application was detected. The effects of SNC on RBCs for 1–24 h (all gates) were similar to t-BOOH at a concentration of 0.5 mM. It is important to underline that t-BOOH induced only MV (gate 4), but not MP (gate 2) formation. Analysis of RBC MCV changes is presented in Figure 3. Low doses of t-BOOH (0.5–1.5 mM) increased cell volume, whereas 2 mM of t-BOOH did not significantly change cell volume. In contrast, A23187 significantly decreased MCV, and SNC had no significant effect on MCV.

### 3.3. Oxidative Stress Induced Hemoglobin Oxidation to Ferryl (Hemichrome) Forms

**Figure 4:**
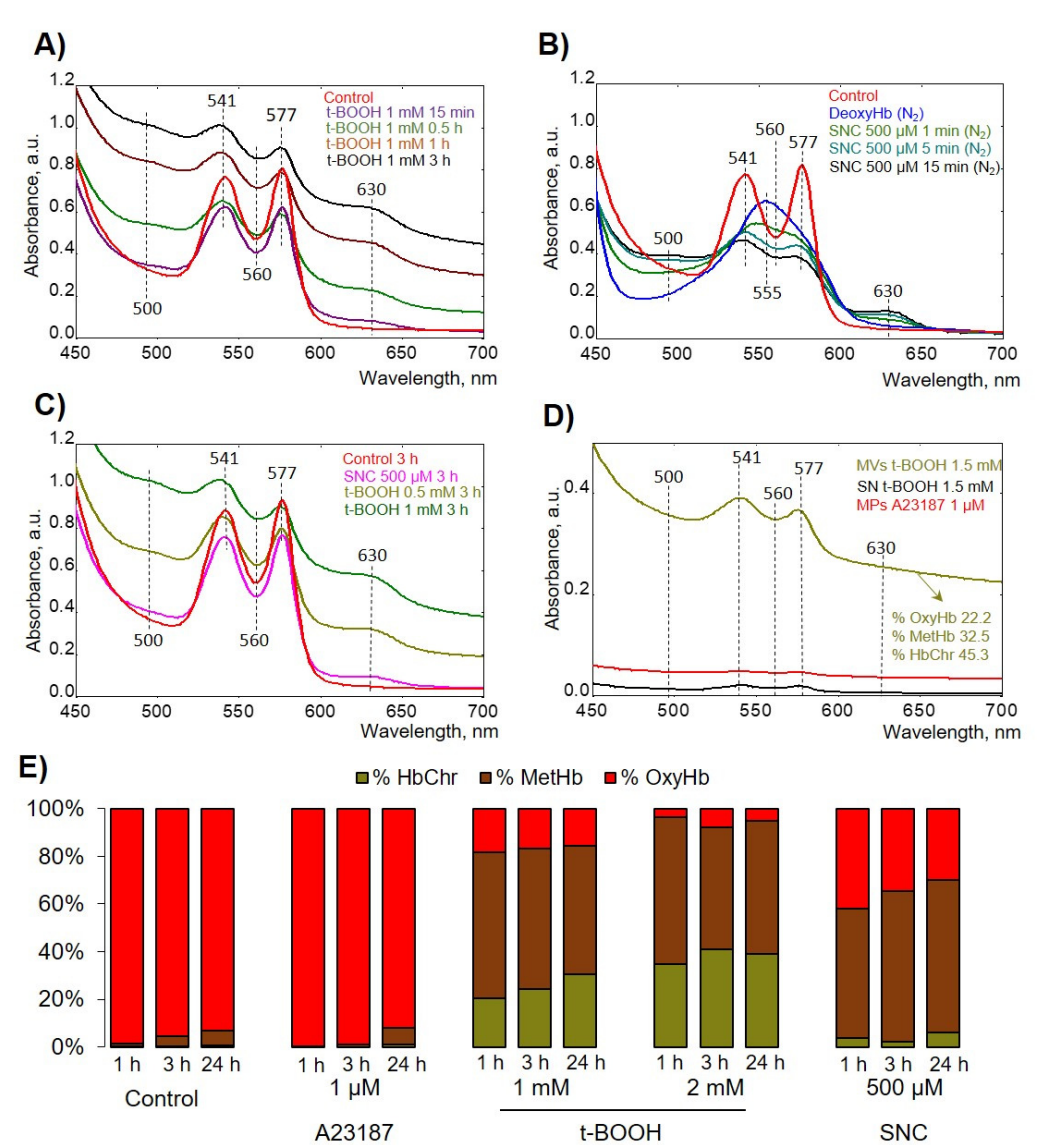
t-BOOH induced hemichrome (HbChr) formation. Spectral scans from 450 to 700 nm captured the different oxidation states of hemoglobin (Hb) identified by characteristic peaks in the visible region. (**A**) Representative spectra of free Hb oxidation by 1mM t-BOOH (ferric, 500 and 630 nm; ferryl/HbChr, 545 nm, 576 nm, and a flattened region between 600 and 700 nm) in comparison with intact Hb spectra (ferrous, 541 and 576 nm) in HEPES-buffer at 25 °C in kinetics. (**B**) Representative spectra of free Hb oxidation by 500 µM SNC in deoxygenated by N_2_ HEPES buffer at 25 °C in kinetics. Free oxyHb was deoxygenated by N_2_ and then SNC was added for the indicated time. (**C**) Spectra of Hb from hypoosmotically lysed RBCs after 3 h treatment with indicated compounds at indicated concentrations. (**D**) After 24 h of RBC incubation with indicated compounds, we collected the MVs and MPs, as described in the Materials and Methods section, and then MVs/MPs and supernatant (SN) from the last washing step were analyzed. (**E**) Representative bar chart of Hb species calculated from one donor.

To evaluate how Hb oxidation state affects formation of RBC-derived MVs and MPs, we first tested the effects of used compounds on different Hb species formation. To maintain maximum oxygen-carrying capacity, Hb must be kept under reducing conditions in the ferrous (Fe^2+^) form by an efficient enzymatic machinery [34]. HbFe^2+^ could spontaneously and during OS be oxidized to form HbFe^3+^ (ferric Hb, methemoglobin, metHb), HbFe^4+^ (ferryl Hb), and ⋅HbFe^4+^ (ferryl radical). These oxidatively unstable Hb intermediates (ferryl/ferryl protein radicals refer to as hemichromes, HbChr) oxidize residues within the Hb globin chains (and other proteins within proximity), ultimately leading to Hb degradation and heme loss [35]. We used several compounds that can oxidize Hb and we elucidated their effects on Hb oxidation state (Figure 4).

First, we tested the effects of used compounds on free Hb (Figure 4A,B), and then intact cells were oxidized and lysed in hypoosmotic conditions, and corresponding spectra were scanned (Figure 4C). t-BOOH firstly oxidized Hb to MetHb, followed by oxidation to HbChr (Figure 4A). To evaluate the effects of NO donor (SNC), we used hypoxic conditions by application of N_2_. SNC first formed HbNO and then was oxidized to MetHb without significant formation of HbChr (Figure 4B,C,E and Table 1). MPs and MVs were collected from RBCs after 24 h of the application of indicated compounds and then supernatants and MV- and MP-containing pellets were scanned spectrophotometrically (Figure 4D). t-BOOH-induced MVs contained ferryl forms of Hb, whereas SNC- and A23187-produced MPs did not contain any significant amount of Hb. The percentage of Hb species was calculated according to [25] and is presented in Figure 4E and Table 1. These data indicated that only t-BOOH-induced oxidative stress resulted in HbChr formation, SNC led only to metHb formation, and increase of intracellular calcium concentration had no effects on Hb oxidation.

### 3.4. t-BOOH Dose-Dependently Decreased RBC Deformability

Next, to characterize OS-induced RBC transformations and possible deformability changes in conditions favorable for MVs and MPs release, we used the osmotic fragility test and ammonium stress test. The osmotic fragility of RBCs is a composite index of their shape; hydration; and, within certain limitations, proneness to in vivo destruction [36,37]. In standard OFT, the percent of hemolysis in increasingly hypotonic solution (0.75%, 0.65%, and 0.60%) is recorded spectrally by optical density. We developed the original automated, easy method for analysis of cells osmotic fragility. Previously, for analysis of band 3 function, we established the original ammonium stress test that is based on band 3 - Rhesus Associated Glycoprotein (RhAG) -facilitated ability of RBCs to swell and lyse in isoosmotic ammonium buffer (NH_4_^+^ buffer) [27,29]. The basic principles of both tests are described in the Materials and Methods section (Section 2.2.5) and Figures S1 and S2.

**Figure 5:**
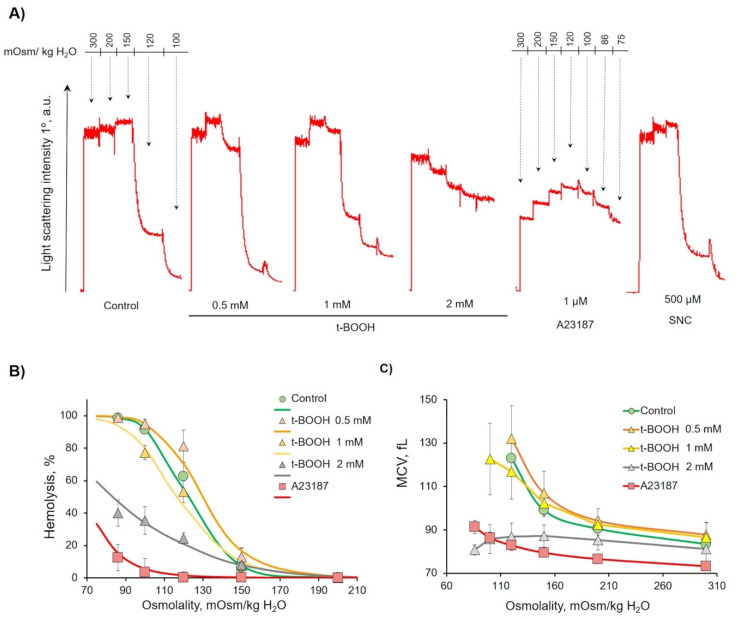
Oxidative stress (OS)-induced decrease in osmotic fragility of RBCs. RBCs (0.5 × 10^9^ cells/mL) were incubated with the indicated substances for 1h, and then aliquots (10 µL, 10^6^ cells/mL final concentration) of samples were resuspended in HEPES buffer with EGTA to register light scattering intensity corresponding to control. Then, the osmolality was gradually reduced by H_2_O supplementation, from 300 to 70 mOsm/kg H_2_O, to maintain the RBC concentration, and the corresponding number of cells was added at each step of H_2_O supplementation. (**A**) Representative osmotic hemolysis curves from the osmotic fragility test (OFT). (**B**) Quantification of percentage of hemolysis from osmotic fragility test calculated from six independent experiments. (**C**) Quantification of MCV during osmotic fragility test calculated from six independent experiments.

The 1 h t-BOOH (0.5–1 mM) treatment led to an increase in H_50_ (the osmolality that triggers hemolysis of 50% cells), indicating elevated fragility, and high doses (2 mM) decreased H_50_, indicating increased rigidity (Figure 5A) in comparison with the control cells. OS dose-dependently increased cell distribution width (W). Both parameters strongly indicated the significant decrease in RBCs’ deformability and increase in RBCs’ heterogeneity.

After 1 h of treatment, A23187 led to a significant decrease of MCV_300_ and subsequent MCV_120_, as well as a significant decrease of H_50_, indicating decreased deformability. SNC effects were similar to 0.5 mM of t-BOOH, MCV_300_ and MCV_120_, W, and H_50_ were slightly increased in comparison to control cells. The osmotic fragility test parameters are summarized in Table 2.

**Figure 6:**
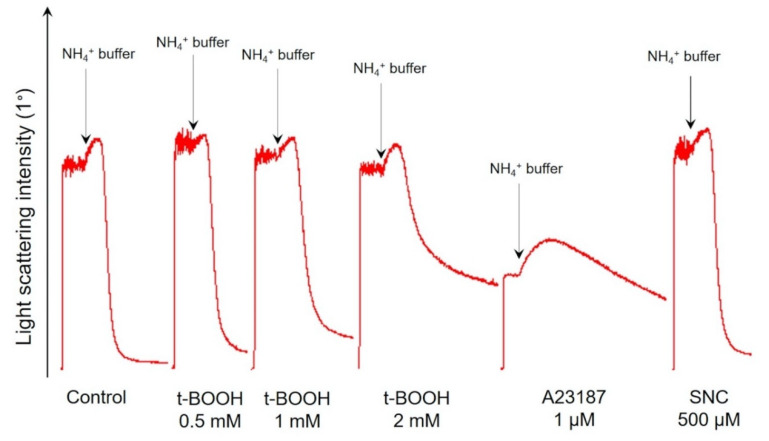
OS dose-dependently decreased RBCs’ deformability and inhibited band 3 function. Representative hemolysis curves of ammonium stress test one of eight experiments. RBCs (0.5 × 10^9^ cells/mL) were incubated with the indicated compounds for 1 h, and then aliquots (10 µL, 10^6^ cells/mL final concentration) of samples were resuspended in HEPES buffer to register light scattering intensity corresponding to control. Then aliquots (10 µL, 10^6^ cells/mL final concentration) of samples were resuspended in NH_4_^+^ buffer for ammonium stress test. Arrows indicate the start of the ammonium stress test. Quantitation of these data is presented in Table 3.

Ammonium stress test results after 1 h were similar to the osmotic fragility test (Figure 6); quantification of ammonium stress test data is presented in Table 3. %Hem (percentage of hemolyzed cells), V_hem_ (hemolysis maximal rate), and MCV_hem_ (maximal mean cell volume during the ammonium stress test) as the markers of rigidity dose-dependently significantly decreased with the rise in t-BOOH concentration, indicating the increased RBC rigidity and inhibited band 3 protein activity.

As described [38,39], an increase in intracellular calcium concentration significantly altered RBCs’ deformability. In our experiments, RBC treatment with A23187 led to a significant decrease in V_hem_, %Hem, and MCV_120_, indicating the increased RBC rigidity. SNC did not cause any significant deformability alterations compared to control; its effects were similar to those triggered by 0.5 mM of t-BOOH.

Results of both important tests characterizing RBC features revealed that t-BOOH dose-dependently decreased RBCs deformability, accrual of intracellular calcium concentration increased RBC rigidity, and SNC had no significant effect on these parameters.

### 3.5. OS Induced RBC Transformation and Microvesicle Formation Was Independent on Extracellular Calcium Concentration

**Figure 7:**
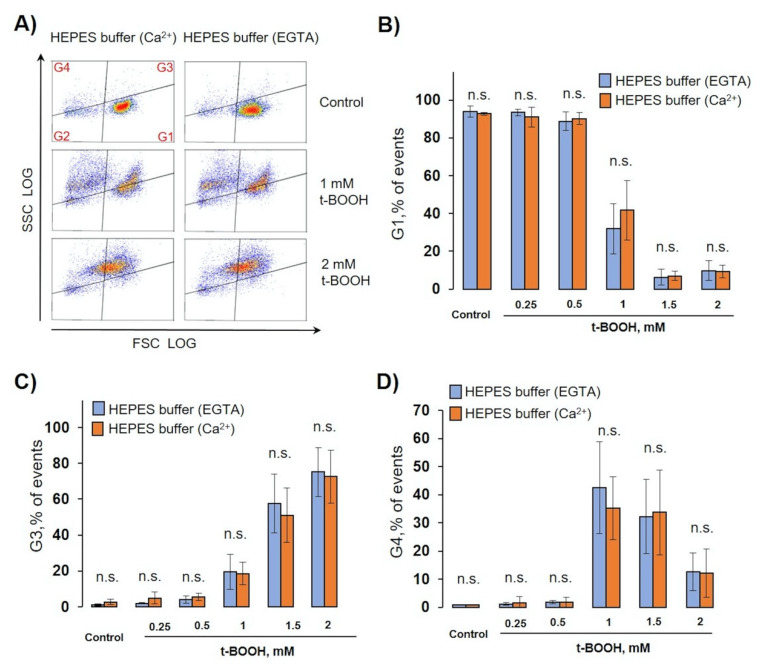
OS-induced RBC transformation and MV formation were calcium-independent. RBCs (0.5 × 10^9^ cells/mL) were incubated with the indicated concentration of t-BOOH in HEPES buffer containing 2 mM calcium, or 2 mM EGTA, for indicated times and were analyzed by flow cytometry. (**A**) Representative SSC/FSC dot plots of one out of six independent experiments for 24 h. Template and gating correspond to Section 3.2. (**B**–**D**) Calculation of events distributed in the corresponding gates. Data are presented as mean ± SD (*n* = 7), paired *t*-test; n.s., not significant.

**Figure 8:**
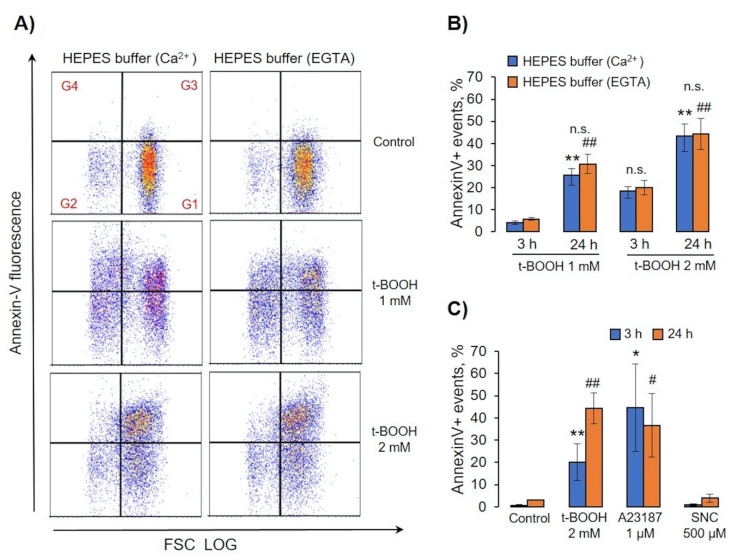
OS-induced annexin-V binding was calcium-independent. RBCs (0.5 × 10^9^ cells/mL) were incubated with indicated concentrations of t-BOOH, A23187, and SNC in HEPES buffer containing 2 mM Ca^2+^ or 2 mM EGTA for indicated times. Annexin-V (0.1 µg/mL, 15 min, 25 °C) was added to treated cells and analyzed by flow cytometry. (**A**) Representative annexin V/FSC dot plots of one out of six independent experiments for 24 h. Gate G1 corresponds to control cells; G2 to annexin-V-negative EVs; G3 to annexin-V-positive cells; G4, annexin-V-positive EVs. (**B,C**) Calculation of annexin-V-positive events in G3 and G4. Data in (**B**–**D**) are presented as the mean ± SD, in (**B**)—paired *t*-test; ** *p* < 0.001, compared to 3h in HEPES buffer with Ca^2+^; ## *p* < 0.001, compared to 3 h in HEPES buffer with EGTA; n.s.—not significant. In (**C**)—one-way ANOVA, Levene’s test < 0.05, Tamhane’s T2 post hoc; * *p* < 0.05, ** *p* < 0.001, compared to 3 h control; # *p* < 0.05, ## *p* < 0.001, compared to 24 h control.

**Figure 9:**
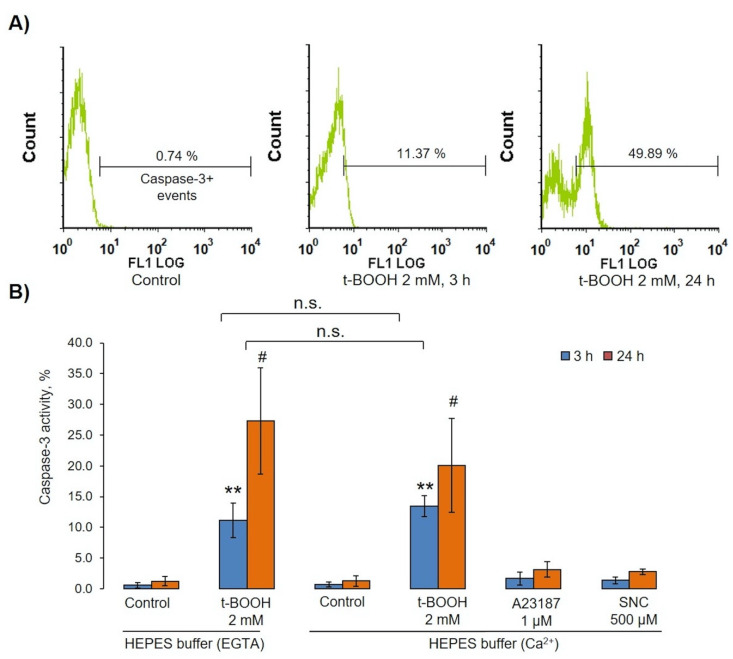
t-BOOH-induced OS activated caspase 3 in RBCs. RBCs (0.5 × 10^9^ cells/mL) were incubated with indicated concentrations of t-BOOH, A23187, and SNC in HEPES buffer containing 2 mM calcium or 2 mM EGTA for indicated times. After we incubated them with indicated compounds, cells were fixed by 1% (final concentration) of methanol-free formaldehyde, permeabilized by 0.5% Tween for 20 min, and then anti-active caspase-3 antibodies were added for 30 min, with caspase-3 activation being measured by flow cytometry according to the manufacturer’s instructions. (**A**) Original histograms from one of seven independent experiments. (**B**) Data quantification based on seven independent experiments. Data are presented as the mean ± SD (*n* = 7), one-way ANOVA (HEPES buffer with EGTA both 3 h and 24 h), Tukey HSD post hoc (HEPES buffer with Ca^2+^ 3 h), Tamhane T2 post hoc (HEPES buffer with Ca^2+^ 24 h); paired t-test HEPES buffer (EGTA) and HEPES buffer (Ca^2+^), ** *p* < 0.001 compared to corresponding control, # *p* < 0.05 compared to corresponding control, n.s.— not significant.

Next, we tested whether extracellular calcium plays any role in OS-induced MP or MV formation. It is well known that calcium plays a significant role in RBCs function [40,41,42] and that it triggers eryptosis [43]; however, the role of calcium in MV formation is not yet clear. All our experiments were performed in both HEPES buffer with EGTA or with 2mM calcium. In normal conditions (intact RBCs, Ca^2+^ - enriched buffer) intracellular calcium concentration maintains less than 60nM [40,44], whereas addition of A23187 equals intra- and extracellular concentrations (2mM) with the following change in features of RBCs [45,46]. Therefore, we compared MV formation, PS surface exposure, and caspase-3 activation in RBCs during OS induced by different t-BOOH concentrations, A23187, and SNC, in HEPES buffer with calcium or with EGTA (Figure 7, Figure 8 and Figure 9).

For MV formation analysis, we used the same template as in Section 3.2 and calculated events in gates G1, G3, and G4 in HEPES-buffer containing calcium (2 mM) or EGTA (2 mM). Surprisingly, calcium had no significant effect on OS-induced RBC transformations and MV formation (Figure 7A–D). Next, we tested whether PS surface exposure (annexin-V binding) as a marker of eryptosis is dependent on extracellular calcium. Similar to MV formation, calcium had no significant effect on OS-induced annexin V binding (Figure 8A,B). We compared percentage of annexin V-positive cells after A23187, t-BOOH, and SNC treatment (Figure 8C). A23187 and t-BOOH after 3 h significantly increased PS exposure, whereas SNC had no significant effect.

In different conditions, PS surface exposure might be dependent, or independent, upon caspase-3 activation [47,48]. t-BOOH induced strong caspase-3 activation (Figure 9A,B), which, like MV formation and PS surface exposure, was independent of calcium. In all tested conditions, A23187 and SNC did not significantly activate caspase-3 (Figure 9B).

### 3.6. t-BOOH-Induced OS Triggered Band 3 Clustering

**Figure 10:**
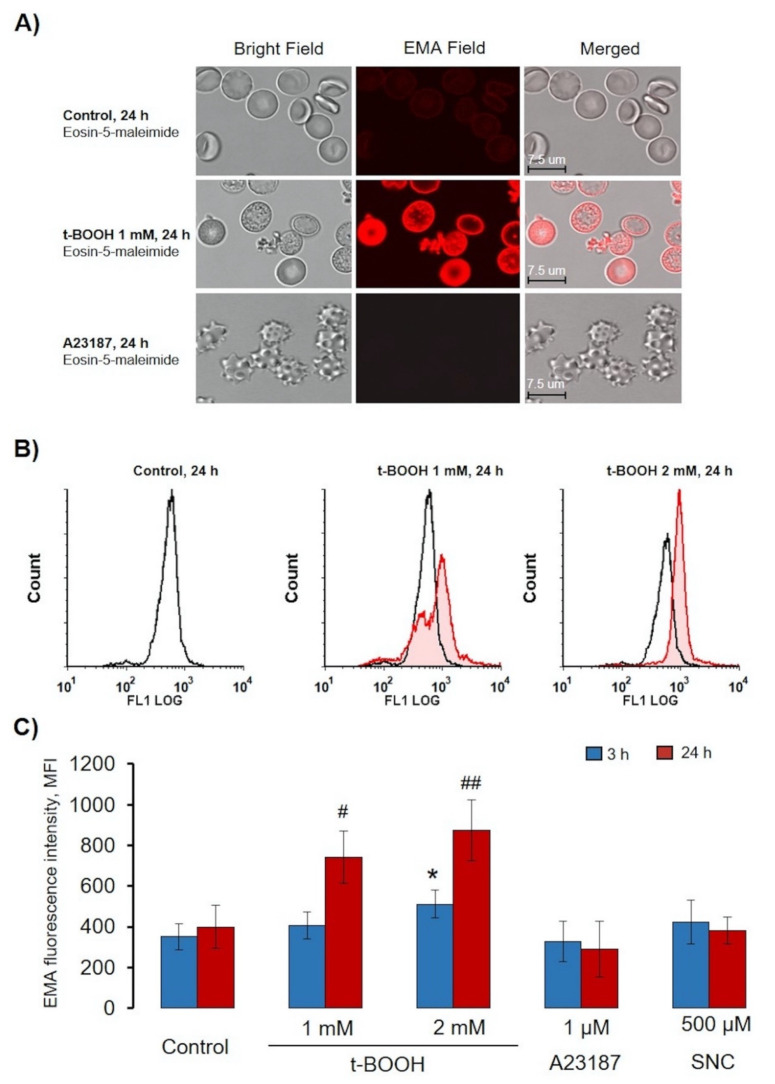
t-BOOH-induced oxidative stress led to band 3 clustering and MV formation. RBCs (0.5 × 10^9^ cells/mL) were incubated as indicated with t-BOOH, A23187, and SNC at the indicated times, followed by EMA staining (0.07 mM, 40 min). (**A**) Representative confocal images of t-BOOH transformed RBCs. RBCs were processed for confocal microscopic analysis as described in the Materials and Methods section (Section 2.2.7). (**B**) Original histograms of EMA fluorescence intensity after 24 h t-BOOH treatment. (**C**) Quantification of flow cytometry data. Data are presented as mean ± SD (*n* = 7), one-way ANOVA, Levene’s test > 0.05, Tukey HSD post hoc (3 h) was used, * *p* < 0.05, compared to 3 h control; Levene’s test < 0.05, Tamhane T2 post hoc (24 h) was used, # *p* < 0.05, ## *p* < 0.001 compared to 24 h control.

Band 3 plays a significant role in RBC functions, and clustering of this protein was demonstrated in several RBCs disorders [7,21,23]. Additionally, band 3 clustering is one of important signals to eliminate senescent and pathological RBCs [49]; therefore, we next used an EMA test to elucidate the effects of the used compounds on band 3 clustering. Oxidative stress induces band 3 oxidation and dissociation from spectrin skeleton, resulting in enhanced mobility and subsequent band 3 cluster formation [21,23,49]. In our experiments, we tested whether each of the used compounds triggers band 3 clustering by applying the EMA-binding test. As expected, in control cells, EMA showed slight homogeneous fluorescence (Figure 10A and Figure S4). During t-BOOH-induced OS, in both RBCs and RBC-derived MV, we detected clustering of band 3 (Figure 10A and Figure S4). In contrast, A23187 and SNC induced neither MV formation nor band 3 clustering (Figure 10C and Figure S4), and, as expected [50,51], A23187 triggered the formation of narrow spikes on the outer half of the bilayer, the so-called echinocytosis, in RBC (Figure 10A and Figure S4).

## 4. Discussion

Extracellular vesicles (EVs) released by different cell types play a significant role in many physiological and pathophysiological processes. The cargo and types of EVs and their functional role strongly depend on the cell of origin and mechanisms of EV formation [52]. It was reported that RBC-derived EVs carry residual Hb, lipids, and proteins that are in charge of known physiological effects of EVs [53,54]. RBC-derived EVs were shown to express phosphatidylserine and cell-specific band 3 epitopes on the surface, as well as to contain enzymes involved in redox homeostasis (glutathione S-transferase, ubiquitin, thioredoxin, peroxiredoxin-1, peroxiredoxin-2) and complement-inhibiting proteins CD55 and CD59. The EV effects in health, disease, and blood transfusion are a matter of continued investigation [3,15,54,55,56,57,58]. However, it has already been defined that MVs could be captured by other circulated blood cells and by endothelial cells; thereby, EVs are involved in coagulation promotion, inflammation, immune modulation, endothelial dysfunction, and vasodilatation impairment, as well as having vasoactive properties potentially altering oxygen delivery homeostasis [17]. Moreover, EVs from RBCs could increase production of tumor necrosis factor-alpha in monocytes and CD4+, as well as CD8+ T-cell proliferation [57].

Two main mechanisms of RBC-derived EV formation connected with an increase of intracellular calcium concentration and Hb oxidation were proposed [58]. The increase in intracellular calcium concentration leads to programmed cell death, so-called eryptosis for RBCs [59], which could be triggered by numerous xenobiotics and exogenous substances [60] as well as several pathological states including diabetes, hepatic failure, sepsis, and chronic kidney disease [61,62,63,64,65]. As expected [38,39,41], intracellular calcium increase mediated by A23187 led to RBC shrinkage (Figure 3 and Figure 5) and subsequent rapid loss of flexibility (Figure 5 and Figure 6), which could be explained by the opening of the Ca^2+^-sensitive Gardos channels resulting in hyperpolarisation and a loss of K^-^, Cl^-^, and water [66]. MPs formed during eryptosis by membrane shedding are around 200 nm in diameter and are characterized by surface phosphatidylserine (PS) exposure [9]. However, the question of whether EV formation during eryptosis is connected with classical apoptotic pathways (caspase-3 activation) is still under debate [7,13]. In our experiments, increase in intracellular calcium mediated by application of A23187 led to strong PS surface exposure in both RBCs and EVs but did not activate caspase-3 (Figure 9B). In contrast, OS-triggered PS exposure and EV formation were independent of calcium and caspase-3 activation, which indicated that two different mechanisms, calcium-dependent and -independent mechanisms, are responsible for surface PS exposure and EV formation. The classification of RBC-derived EVs is also not yet clearly defined. Some authors refer to these as microvesicles [13,17], while others use the term microparticles for the same kind of EVs [2,3], without detailed characterization of differences between them. In our study, we divided RBC-derived EVs into two populations: (a) EV, derived from A23187-treated RBCs, which were smaller and did not contain Hb, referred to as MPs, and (b) OS-triggered EVs, which were bigger and contained oxidized Hb, referred to as MVs [22]. Various compounds and pathological states might unquestionably trigger both pathways of RBC-derived EV formation, however, in our study, we focused mainly on important conditions that facilitate OS-induced RBC transformation, MV formation, and characterization of these MVs.

The increase in intracellular calcium concentration is regarded as the main driving force of RBC transformation and EV formation [17]. OS-induced increase of intracellular calcium mobilization was shown in several publications [40,67], however, the question as to whether it is the main trigger of RBC transformation remained open; therefore, in our experiments, we first tested the influence of extracellular calcium on RBC transformation and EV formation, comparing HEPES buffer with 2 mM calcium with HEPES buffer containing 2 mM EGTA. Surprisingly, we found no significant differences in OS-induced RBC transformation and EV formation in these two conditions. Most of our experiments were performed in HEPES buffer with 2 mM EGTA, and similar experiments were performed in HEPES buffer with 2 mM calcium (Figure 7), with there being no significant differences in RBC transformation, annexin-V binding (Figure 8A,B), MV formation, or caspase-3 activation (Figure 9B). On the basis of these data, we concluded that Hb oxidation to HbChr but not increase of intracellular calcium concentration is most likely the main driving force of OS-induced RBC transformation and EV formation.

The next important question raised in our study was connected with oxidant and RBC concentration, as well as time of incubation with oxidant (Figure 1). In most of the literature, t-BOOH is used to induce OS in RBCs, with highly controversial results, from an increase of cell volume to cell shrinkage, content of produced microparticles, and cell deformability. Here, we showed that RBC transformation, deformability, band 3 clustering, and MV formation in t-BOOH-induced OS strongly depended on oxidant concentration/time and the ratio of oxidant to cell count (Figure 1). t-BOOH concentration less than 0.5 mM did not induce significant changes in RBCs; MV formation started at concentrations 1–1.5 mM, whereas at 2 mM, cells rigidity increased with reduced MV formation (Figure 5). We also checked whether the effects of t-BOOH during long-term treatment are reversible or not, finding that even 30 min t-BOOH application is sufficient to make the effects of oxidant irreversible (Figure S3).

Another aspect of OS-induced RBC transformation that was also an important part of our work and should be considered in future studies was connected with the calcein-AM test, a marker of cellular esterase activity. Clearly, esterase activity did not directly correlate with RBC transformation and MV formation (Figure 1 and Figure 2). t-BOOH dose-dependently reduced calcein fluorescence; however, cell transformation and MV formation continued even in the conditions of almost completely inactivated esterase activity after 3 h of incubation with t-BOOH (Figure 2).

Processes such as senescence, hyperthermia, transfusion, increase of intracellular calcium concentration, RBC storage in blood banks, and oxidative stress accelerate RBC vesiculation [11]. Through MV generation, erythrocytes were shown to remove membrane patches containing removal molecules, damaged cells and membrane constituents [15,17,58]. In our experiments, only t-BOOH induced the formation of high SSC MVs, which contained highly oxidized Hb (HbChr), whereas A23187 and SNC did not, indicating that RBC-derived EVs were heterogeneous in content, with or without Hb. These data indicated that during OS, RBCs eliminate HbChr by vesiculation in order to sacrifice the cell itself, thereby prolonging lifespan and delaying the untimely clearance of in all other respects healthy RBCs [6,9,15].

## 5. Conclusions

OS-triggered RBC transformations and MV formation is mediated by complex processes including Hb oxidation, band 3 clustering, cytoskeleton reorganization, an increase in intracellular calcium concentrations, and other alterations in RBC cellular organization. OS could be developed as consequences of several pathological states such as diabetes, sepsis, chronic kidney disease, and hepatic failure, and the degree of OS and RBC transformations might be significantly variable, being dependent on the conditions. t-BOOH is often used for the analysis of RBCs in OS conditions, however, published data focusing on RBCs’ responses to OS are highly variable and depend on concentration/time of t-BOOH administration. Therefore, we first established standards for evaluation of t-BOOH-induced OS and showed that it is especially important to consider not only oxidant concentration but also the ratio of oxidant to cell count. Next, we developed two new original methods, ammonium stress-test and automated osmotic fragility test, based on laser diffraction analysis of RBC transformations, which allow us to characterize RBCs’ osmotic and ammonium fragility. Thus, the presented data, methodology, and new methods for the analysis of OS-induced RBC transformations will allow us to characterize these cells more broadly during OS.

## Figures and Tables

**Table 1:** The percentage of Hb species formation in response to indicated compounds calculated according to Kanias et al. [25].

Hb Species	Control		t-BOOH						A23187		SNC	
			0.5 mM		1 mM		2 mM		1 µM		500 µM	
	1 h	3 h	1 h	3 h	1 h	3 h	1 h	3 h	1 h	3 h	1 h	3 h
% OxyHb	98.0 ± 2.0	95.6 ± 3.0	66.3 ± 4.2 *	45.3 ± 3.9 *	30.7 ± 9.0 **	27.0 ± 13.6 **	20.9 ± 16.4 **	20.7 ± 15.1 **	97.4 ± 3.1	96.6 ± 3.0	47.2 ± 5.9 **	33.1 ± 3.4 **
% MetHb	1.8 ± 0.1	4.0 ± 2.3	30.6 ± 5.1 *	49.1 ± 6.6 *	50.5 ± 8.1 **	47.0 ± 11.8 **	58.8 ± 9.4 **	46.1 ± 6.6 **	2.3 ± 0.5	3.4 ± 0.6	50.8 ± 4.1 **	63.4 ± 4.6 **
% HbChr	0.2 ± 0.6	0.4 ± 0.2	4.9 ± 3.6	5.4 ± 2.1	18.8 ± 5.4 *	25.9 ± 8.1 **	20.3 ± 12.8 *	33.2 ± 9.1 **	0.3 ± 0.2	0.2 ± 0.1	2.5 ± 1.4	3.6 ± 2.5

Data are presented as means ± SD (*n* = 7), one-way ANOVA, Levene’s test < 0.05, and Tamhane T2 post hoc. * *p* < 0.05, ** *p* < 0.001, compared to corresponding control (1 h or 3 h).

**Table 2:** Quantification of osmotic fragility test (OFT) data of six independent experiments (donors).

OFT Characteristics	Control	t-BOOH			A23187	SNC
		0.5 mM	1 mM	2 mM	1 µM	500 µM
H_50_, mOsm/kg H_2_O	122 ± 9	130 ± 7	117 ± 8	83 ± 6 **	68 ± 7 **	128 ± 6
W, mOsm/kg H_2_O	44 ± 7	52 ± 8	60 ± 9 *	107 ± 10 **	41 ± 6	47 ± 7
MCV_300_, fL	84 ± 2	88 ± 6	87 ± 7	81 ± 6	73 ± 2 **	86 ± 5
MCV_120_, fL	124 ± 9	131 ± 8	115 ± 8	88 ± 6 *	83 ± 2 **	127 ± 4

MCV_300_—data from hematology cell counter. Data are presented as means ± SD (*n* = 6), one-way ANOVA; if Levene’s test < 0.05, Tamhane T2 post hoc (MCV_300_,) was used; if Levene’s test > 0.05, Tukey HCD post hoc (H_50_, W, MCV_120_) was used. * *p* < 0.05, ** *p* < 0.001, compared to corresponding control.

**Table 3:** Quantification of ammonium stress-test data of five independent experiments (donors).

AST Characteristics	Control	t-BOOH				A23187	SNC
		0.5 mM	1 mM	1.5 mM	2 mM	1 µM	500 µM
V_hem_	1.00 ± 0.02	1.01 ± 0.02	0.71 ± 0.21	0.57 ± 0.08 *	0.32 ± 0.09 *	0.06 ± 0.02 **	1.04 ± 0.02
%Hem	96.5 ± 0.2	92.1 ± 2.6	80.8 ± 11.4	69.3 ± 9.6 *	47.8 ± 13.2 **	40.0 ± 11.3 **	95.6 ± 1.0
MCV_300_	84.9 ± 0.9	86.7 ± 2.3	87.5 ± 5.9	87.8 ± 4.3	88.6 ± 10.4	70.5 ± 3.7 *	86.87 ± 1.9
MCV_hem_	142.3 ± 3.2	143.8 ± 4.8	124.5 ± 9.8	116.0 ± 1.7 *	105.1 ± 1.9 **	96.3 ± 6.5 **	141.0 ± 2.8

MCV_300_—data from hematology cell counter. Data are presented as means ± SD (*n* = 5), one-way ANOVA; if Levene’s test < 0.05, Tamhane T2 post hoc (V_hem_, MCV_300_, MCV_120_) was used, if Levene’s test > 0.05, Tukey HSD post hoc (%Hem) was used. * *p* < 0.05, ** *p* < 0.001, compared to corresponding control.

## Supplementary Materials

The following are available online at https://www.mdpi.com/2076-3921/9/10/929/s1, Figure S1: Evaluation of RBCs’ osmotic fragility by laser diffraction method. Figure S2: Basic principles of ammonium stress-test. Figure S3: Effects of 30 min t-BOOH-induced oxidative stress on RBCs were irreversible for 4 and 24 h. Figure S4: t-BOOH-induced oxidative stress led to band 3 clustering and MV formation.
