# Supplementary material for: Microvesicle Formation Induced by Oxidative Stress in Human Erythrocytes
Source: Antioxidants (Basel). 2020 Sep 28;9(10):929. doi: 10.3390/antiox9100929 (PMC7650597; doi:10.3390/antiox9100929)
Supplement: Supplementary file 1 [file antioxidants-09-00929-s001.zip › antioxidants-932456-supplementary.docx]

**Supplementary Information**

**Microvesicle formation induced by oxidative stress in human erythrocytes**

J. Sudnitsyna^1,2^, E. Skverchinskaya^2^, I. Dobrylko^2^, E. Nikitina^2^, S. Gambaryan^2,3^, I. Mindukshev^2^.

**
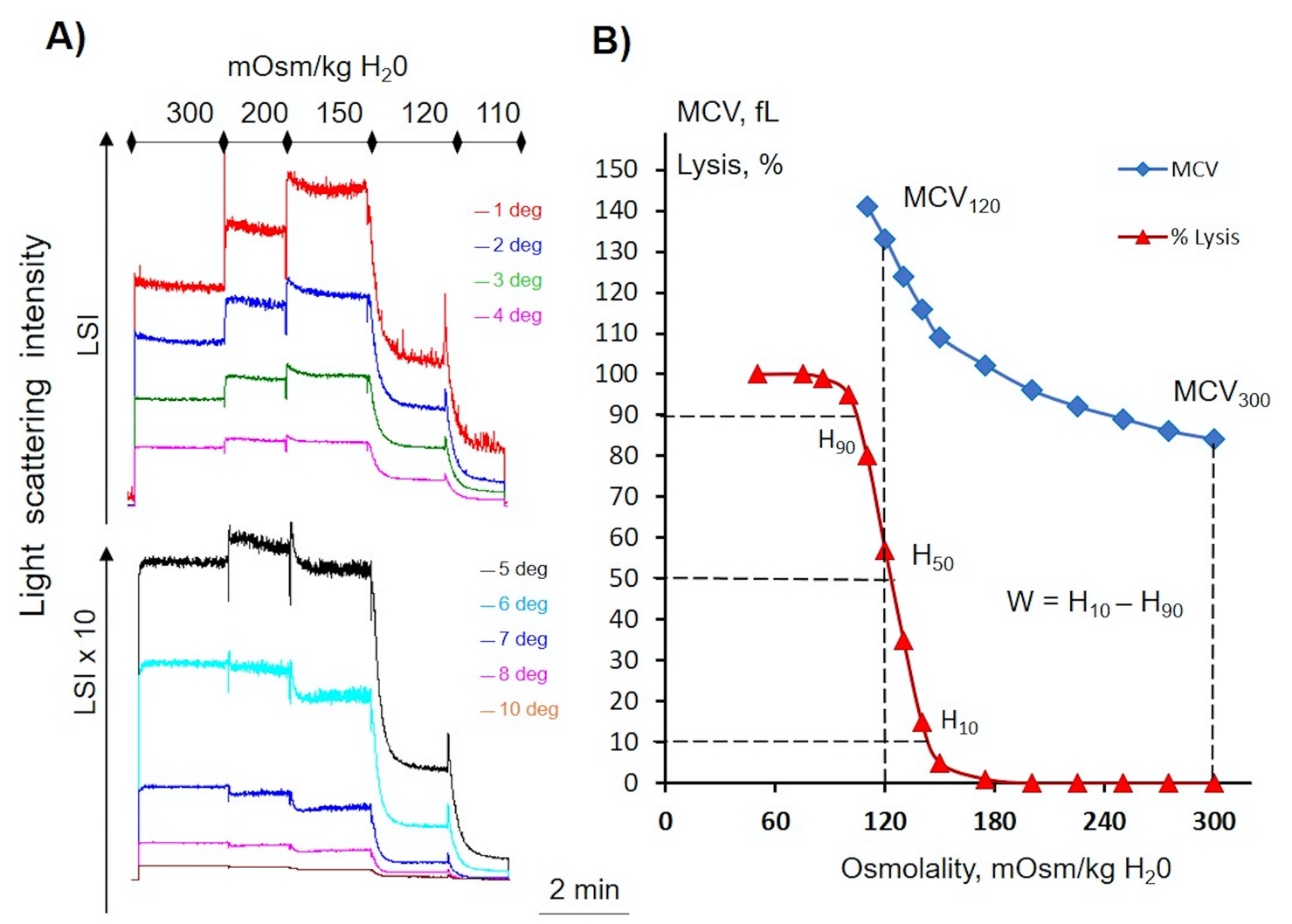
**

**Supplementary Figure 1**. Evaluation of RBC osmotic fragility by laser diffraction method. Light scattered intensity (LSI) was measured from RBC (1x10^6^ cells/ml) in 1 ml HEPES buffer with 2mM EGTA (osmolality 300 mOsm/kg H_2_O) then water and RBCs were added at indicated time to adjust the osmolality to the desired value and to keep RBC concentration constant. (**A**) LaSca-TM analyzer original records of LSI changes at different angles. For the angles from 5° to 10° Y axis is multiplied by 10; (**B**) Dependence of MCV (fL) and % of hemolysis from the buffer osmolality. Initial MCV (MCV_300_) taken from hematologic counter and was used for future calculations of MCV changes by original software LaSca v.1498. MCV_120_ indicates the maximum cell volume during the osmotic fragility test, H_10_, H _50_, H _90_ indicate the osmolality at which 10, 50, and 90% of cells are lyzed. RBC distribution width (W) calculates as W = H_10_ – H_90_ (mOsm/kg H_2_O).

**
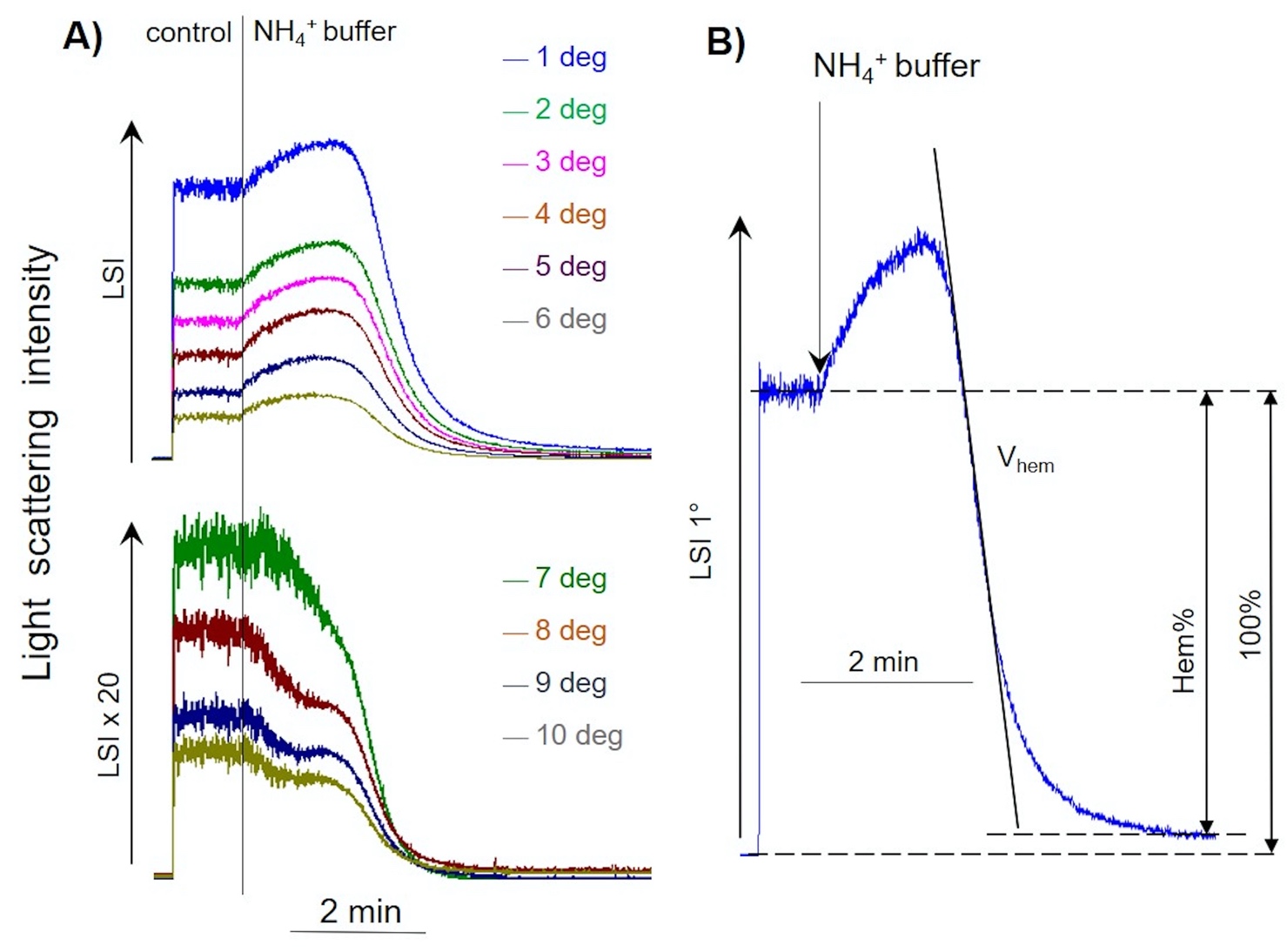
**

**Supplementary Figure 2**. Basic principles of ammonium stress-test. This test was developed based on the unique ability of RBCs to swell to critical values and lyse in isosmotic ammonium buffer (NH_4_^+^ - buffer). Initially light scattered intensity (LSI) was measured from RBCs (1x10^6^cells/ml) in 1 ml HEPES buffer with 2mM EGTA (300 mOsm/kg H_2_O), then the buffer was changed to NH_4_^+^ - buffer (300 mOsm/kg H_2_O). (**A)** Original records of LSI changes at different angles registered by LaSca-TM analyzer. For the angles from 7° to 10° Y axis is multiplied by 10; (**B**) Calculation of % of hemolyzed cells (%Hem) and rate of hemolysis (Vhem) from the hemolysis curves in FS 1°.

**
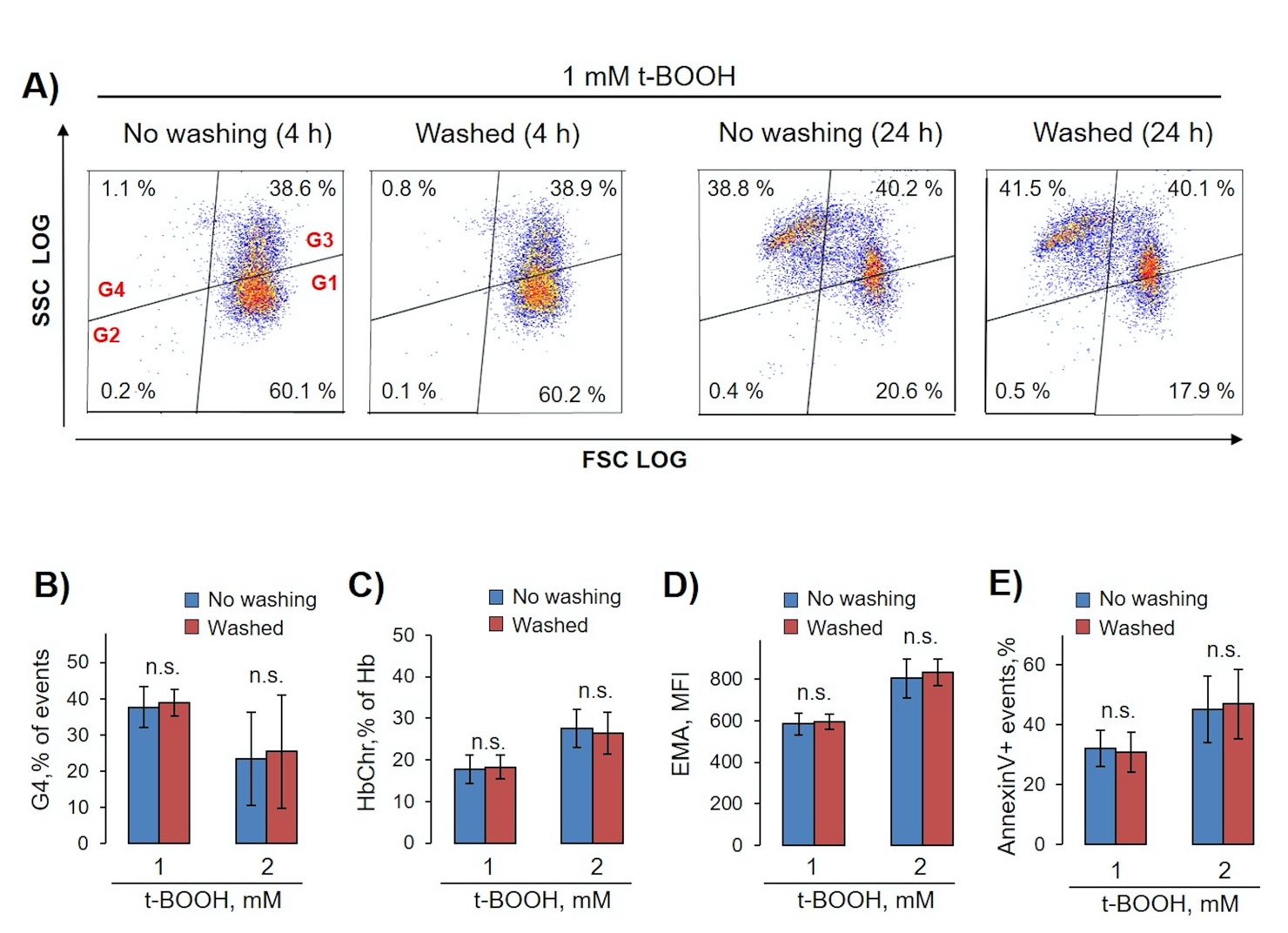
**

**Supplementary Figure 3.** Effects of 30 min t-BOOH-induced oxidative stress on RBCs were irreversible for 4 and 24 h. RBCs (0.5 x 10^9^ cells/mL) were incubated with 1 mM of t-BOOH for 30 min, the cells were divided into two groups, one of which was washed 2 times by HEPES buffer with EGTA (“washed”), and the other left without washing out t-BOOH (“No washing”). Cells were analyzed by flow cytometry as described in Figure 2A. (**A)** Representative dot plots out of six independent experiments. Gate 1 represents control RBCs, gate 2 - MPs, gate 3 - transformed RBCs, and gate 4 – MVs; **(B)** Quantification of events in Gate 4 is expressed as mean ± SD, n=6. (**C**) % of HbChr in RBCs measured spectrophotometrically as described in Figure 4; (**D)** Band 3 clustering analyzed by EMA-test as described in Figure 10; (**E**) Annexin-V binding to RBCs was performed as described in Figure 8; Paired t-test was used to assess significant differences between “washed” and “no washing” samples. P < 0.05 was considered significant, n.s – not significant differences.

**
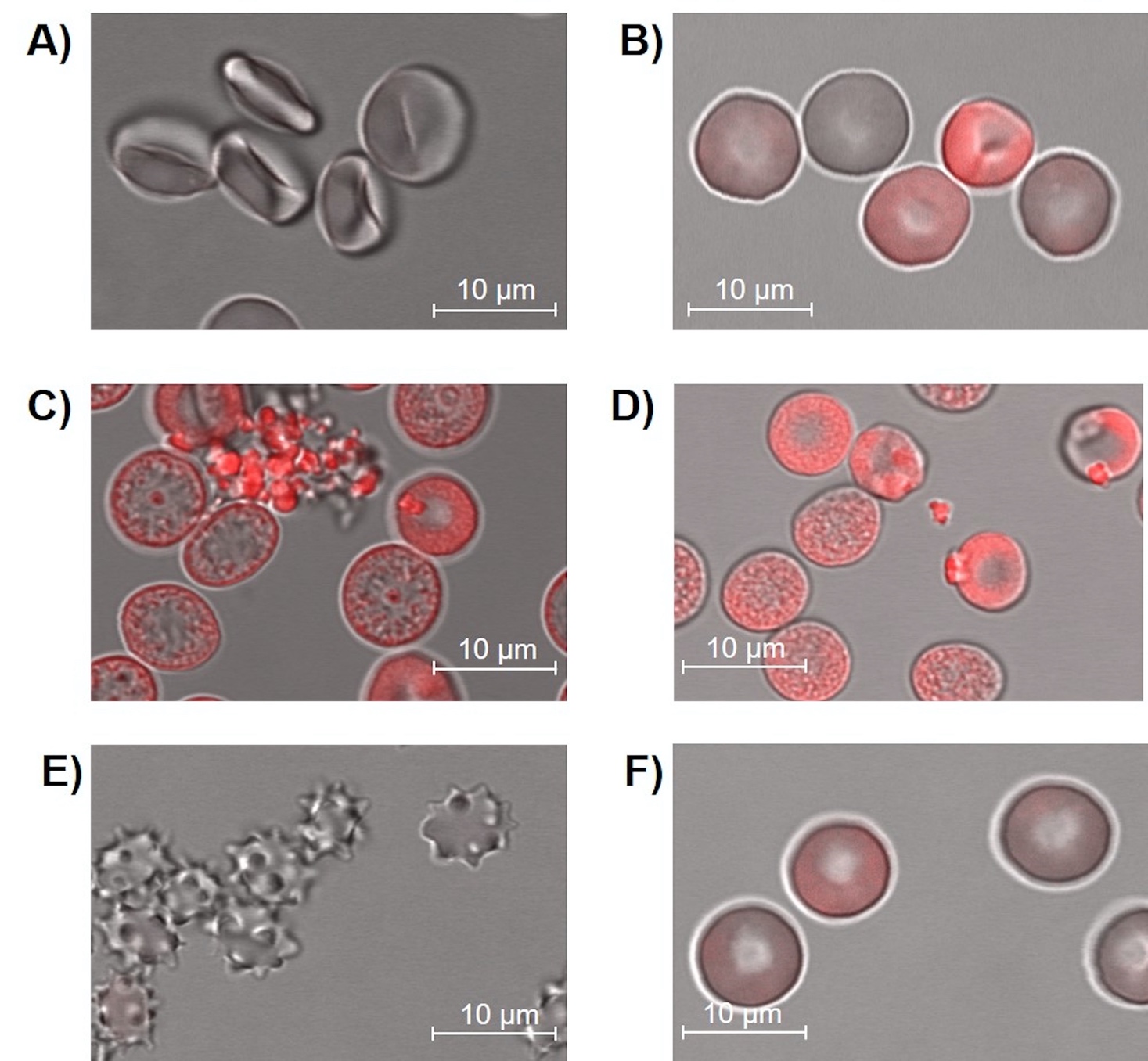
**

**Supplementary Figure 4.** t-BOOH-induced oxidative stress led to band 3 clustering and MV formation. RBCs (0.5x10^9^ cells/mL) were incubated as indicated with t-BOOH, A23187, and SNC, for 24 h, followed by ЕМА staining (0.07mM, 40min), and then were processed for confocal microscopic analysis as described in the Methods part (2.2.7). (**A-F)** representative confocal images of transformed RBCs; (**A)** control RBCs; (**B-D)** RBCs incubated with 0.5, 1, and 2 mM of t-BOOH respectively; (**E**) RBCs incubated with 1 µM of A23187; (**F**) RBCs incubated with 0.5 mM of SNC.
